# Data on Machine Learning regenerated Lithium-ion battery impedance

**DOI:** 10.1016/j.dib.2022.108698

**Published:** 2022-10-28

**Authors:** Selcuk Temiz, Hasan Kurban, Salim Erol, Mehmet M. Dalkilic

**Affiliations:** a Department of Physics, Eskisehir Osmangazi University, Eskisehir, 26040, Turkey; b Applied Data Science Department, San Jose State University, San Jose, CA, 95192, US; c Computer Science Department, Indiana University, Bloomington, 47405, IN, US; d Department of Chemical Engineering, Eskisehir Osmangazi University, Eskisehir, 26040, Turkey

**Keywords:** Machine Learning (ML) on Li-ion batteries, Co-modeling approach, Electrochemical Impedance Spectroscopy (EIS) for Li-ion batteries, Regeration of impedance for Li-ion batteries

## Abstract

This paper describes and provides the data on the regenerated-impedance spectra that is computed from experimental results of electrochemical impedance spectroscopy measurements taken from a commercial Li-ion battery. The empirical impedance data of secondary coin type Li-ion batteries were collected in different states of charge ranging from empty to full state of charge configurations. This approach utilizes only a small seed (*ex grano*) experimental data set to first build an ensemble of weighted disparate models selected based on performance and non-correlative criteria (“co-modelling”) then second to generate what would be the remaining experimental data synthetically. The “Cooperative Model Framework” demonstrates the efficacy of this approach by assessing the synthetically generated data.

**NOMENCLATURE**
EIS: Electrochemical impedance spectroscopy
V_dc_: Potential measured at direct current
I_dc_: Current measured at direct current
f: frequency
Z_phz_: Phase angle of impedance
ML: Machine learning
SoC: State of charge
LIR: Lithium-ion rechargeable
knn: K-nearest neighbor
monmlp: Monotone multi-layer perceptron
svmRadial: Radial support vector machine
gcvEarth: Multivariate adaptive regression splines with generalized cross validation
glm: Generalized linear model
ppr: Projection pursuit regression
rpart: Recursive partitioning and regression tree
rf: Random forest
xgb: Extreme gradient boosting
Dart: Dropouts meet multiple additive regression tree
gausspr: Gauss process
RMSE: Root mean square error
RMSESD: Root mean square error standard deviation
Z_r_: Real part of complex impedance
Z_j_: Imaginary part of complex impedance
dc: Direct current
ac: Alternating current

**Specifications Table**

**Table:** 

Subject	Electrochemical Engineering, Applied Machine Learning, Co-modeling
Specific subject area	Regenerating the impedance spectra of Li-ion batteries by using novel Machine Learning Methods on the results of empirical impedance measurements.
Type of data	Table Image Graph Figure
How the data were acquired	Empirical Impedance data were obtained by using Electrochemical Impedance Spectroscopy set up (Gamry REF3000AE Potentiostat and Echem Analyst) Machine Learning processed data were synthesized by using novel computational techniques on experimental results.
Data format	Raw Analyzed
Description of data collection	The experimental impedance data were collected in cell potentials as 3.2V, 3.4V, 3.6V, 3.8V, 4.0V, and 4.2V (corresponding state of charge values: 0%, 3%, 8%, 40%, 78%, 100%).
Data source location	Experimental impedance data: Institution: Eskisehir Osmangazi University City: Eskisehir Country: Turkey Machine Learning generated impedance data: Institution: Indiana University City/State: Bloomington, Indiana Country: United States of America
Data accessibility	Repository name: Mendeley Data Direct URL to data: https://data.mendeley.com/datasets/hr2ftv6gcn/1 DOI: 10.17632/hr2ftv6gcn.1 Machine Learning Code: https://github.com/hasankurban/Machine-Learning-Based-Regeneration-of-Li-ion-Battery-Impedance
Related research article	S. Temiz, H. Kurban, S. Erol, M. M. Dalkilic, Regeneration of Lithium-ion Battery Impedance using a Novel Machine Learning Framework and Minimal Empirical Data, Journal of Energy Storage, 52 (2022) 105022.

## Value of the Data

• Experimental impedance data taken in particular state of charge (SoC) conditions were used to build a weighted ensemble of disparate models selected from performance and non-correlative criteria.
• The approach is called “The Cooperative Model Framework” (or co-modeling) produce synthetic impedance data from minimal experimental data training.
• The synthetic data can be used on battery management systems in order to monitor the performance of Li-ion batteries.
• The synthetic data can be used to study on the analysis of new battery designs.

## 1 Data Description

The data described in this article were the results of the combination of experimental and machine learning (ML) methods. The experimental impedance data on Li-ion batteries were collected by using electrochemical impedance spectroscopy under different charging conditions. The computational data were obtained by using a novel data-centric machine learning approach to build an ensemble of models to effectively complete the experimental results. The amount of experimental data were intentionally fixed in small volume in order to observe the productiveness of the co-modeling approach with the minimum amount of input.

The impedance data is useful for analyzing and monitoring the characteristics and performance of rechargeable Li-ion batteries. Battery impedance can be represented either by Nyquist or Bode Format according to the purpose of analysis. The empirical data in this article consist of the parameters and results of the offline potentiostatic impedance measurements taken from commercial secondary LIR2032 Li-ion battery cells under different cell potentials from 3.2V to 4.2V. The datasets (3.2, 3.4, 3.6, 3.8, 4, 4.2 .txt files on Mendeley Data) include the excitation potential (V_dc_) and frequency (f) as inputs, and corresponding current (I_dc_) and phase angle of impedance (Z_phz_) as outputs along with the resulting real (Z_r_) and imaginary (Z_j_) components of battery impedance, and these components can be used in Nyquist plotting.

Phase angle of impedance as a function of applied frequency is one of the representations of impedance response of an electrochemical system; and it is designated as the Bode plotting. An equivalent electrical circuit is a useful illustration of an electrochemical system with electrical components in a circuit that provides information about charge and mass transport occurring in the system. The phase angle of impedance for any component in the equivalent electrical circuit corresponding to the electrochemical system is the phase shift between the applied excitation potential and current passing through that component. A novel ML approach (co-modelling approach) is used to process the empirical impedance data in order to regenerate the phase angle of impedance [1]. ML regenerated impedance data for I_dc_ and Z_phz_ (ML-regenerated-impedance-data.txt) is included in the same data repository.

## 2 Experimental Design, Materials and Methods

**Fig. 1:**
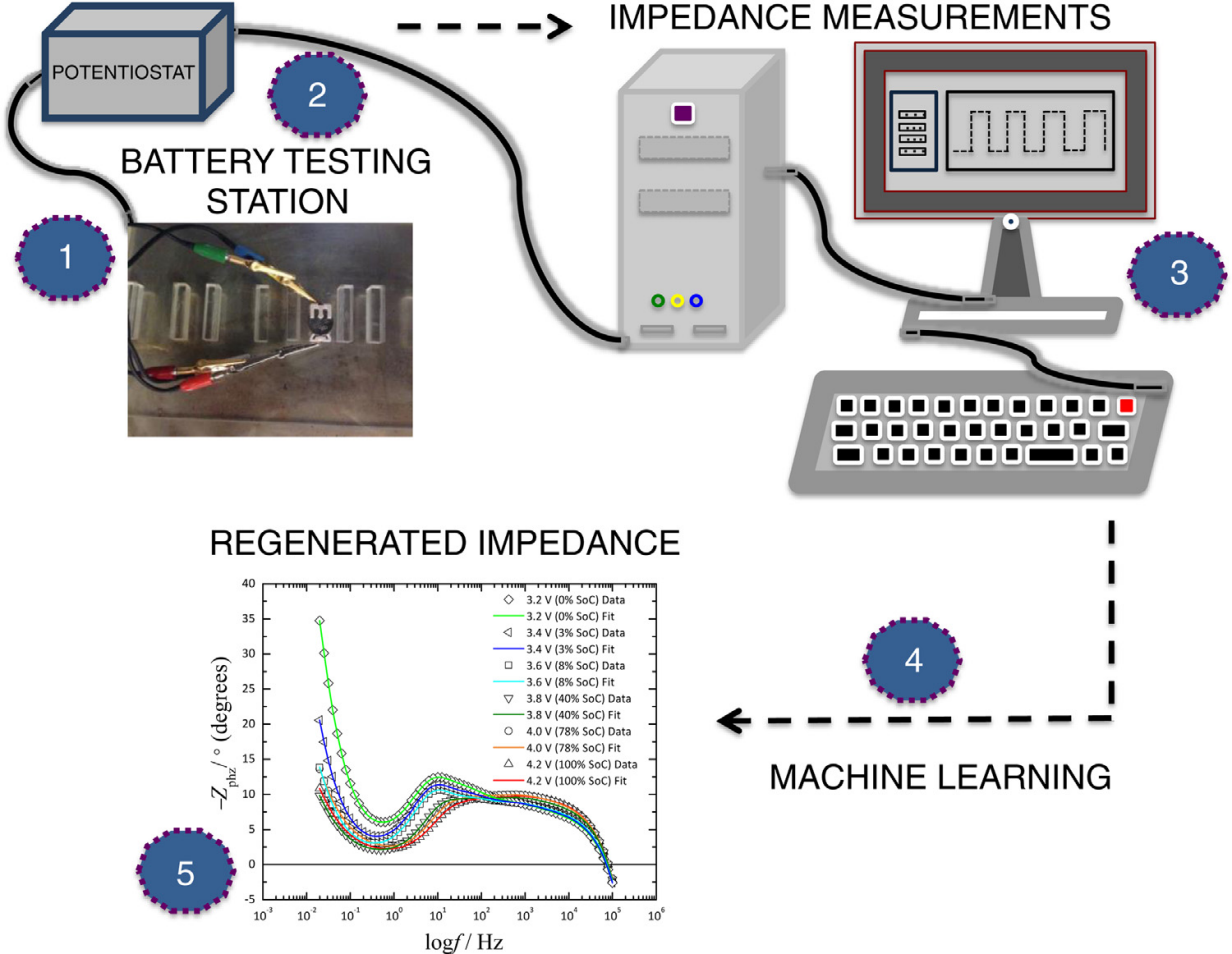
Procedure for battery impedance dataset.

Electrochemical Impedance Spectroscopy (EIS) experiments were performed on commercial coin type Li-ion batteries (LIR2032) using Gamry REF3000AE potentiostat. EIS measurements were taken at different SoC by applying 10 mV perturbation potential between the frequency range of 100 kHz and 10 mHz. SoC is a function of open circuit cell potential of the batteries such that it can be varied by changing the potential of the batteries. A potentiostat can both supply direct or alternating current to the system that is analyzed. The potentiostat provided direct current (dc) to change the cell potential; on the other hand, an alternating current (ac) is applied to measure the impedance at a specific SoC for the batteries. The results of EIS measurements were used to build the co-model. Fig. 1 illustrates the step-by-step procedure for battery impedance dataset; (1) a proper test station for a commercial Li-ion coin cell battery is prepared. The battery is then initially run for one fully charge/discharge cycle prior to impedance measurement; (2) impedance is measured while the battery cell potentials are 3.2V, 3.4V, 3.6V, 3.8V, 4.0V, 4.2V; (3) stored data is analyzed for quality; (4) the co-model is trained and tested then generates remaining impedance; (5) the impedance spectra (consisting of the phase angle as a function of logarithmic frequency) are assessed for goodness of fit.

The details of our Z_phz_ and I_dc_ cooperative models are given in Table 1. Each of these two ensemble models is a weighted ensemble of disparate ML models. The fit of each individual ML model to each cooperative model is demonstrated using RMSE (Root Mean Square Error) and RMSESD (Root Mean Square Error Standard Deviation) values. The last column shows the importance of each model in the co-models.

**Table 1:** The contents of our trained I_dc_ and Z_phz_ cooperative models. e is the scientific e, i.e., 1.04e-04 = 1.04 *×* 10^−4^.

I_dc_ Cooperative Model			
Method	RMSE	RMSESD	Weight
glm	1.04e-04	1.15e-05	-0.102
rf	2.86e-05	1.39e-05	0.389
xgbDART	9.59e-05	2.30e-05	-0.063
xgbLinear	9.98e-05	2.21e-05	-0.080
monmlp	3.35e-05	5.18e-06	0.360
gcvEarth	5.44e-05	1.06e-05	-0.18
ppr	4.14e-05	1.06e-05	0.086
svmRadial	8.07e-05	2.86e-05	0.050
knn	2.99e-05	8.25e-06	0.433
gaussprLinear	1.09e-04	9.75e-06	0.166
rpart	1.12e-04	1.47e-05	-0.072

Z_phz_ Cooperative Model			

xgbLinear	0.6253	0.4062	0.267
xgbDART	0.5301	0.2101	0.374
xgbTree	0.5462	0.1675	0.388

The datasets are available on Mendeley Data.

Code, ML models and ML regenerated impedance are accessible at: https://github.com/hasankurban/Machine-Learning-Based-Regeneration-of-Li-ion-Battery-Impedance.

## Ethics Statements

This work does not contain any human studies or animal experiments. The data were not collected using social media platforms. The authors declare that they have followed the general ethics rules for scientific research and publishing.

## CRediT Author Statement

**Selcuk Temiz:** Conceptualization, Methodology, Writing – original draft preparation, Investigation; **Hasan Kurban:** Conceptualization, Methodology, Writing – original draft preparation, Data curation, Applying Machine Learning algorithms; **Salim Erol:** Conceptualization, Methodology, Writing – original draft preparation, Visualization, Investigation; **Mehmet M. Dalkilic:** Conceptualization, Methodology, Applying Machine Learning algorithms, Writing – review & editing, Supervision.

## Declaration of Competing Interest

The authors declare that they have no known competing financial interests or personal relationships that could have appeared to influence the work reported in this paper.
